# DegS regulates the aerobic metabolism of *Vibrio cholerae* via the ArcA-isocitrate dehydrogenase pathway for growth and intestinal colonization

**DOI:** 10.3389/fcimb.2024.1482919

**Published:** 2024-11-01

**Authors:** Jiajun Zhao, Xiaoyu Huang, Qingqun Li, Fangyu Ren, Huaqin Hu, Jianbo Yuan, Kaiying Wang, Yuanqin Hu, Jian Huang, Xun Min

**Affiliations:** ^1^ Department of Laboratory Medicine, Affiliated Hospital of Zunyi Medical University, Zunyi, Guizhou, China; ^2^ School of Laboratory Medicine, Zunyi Medical University, Zunyi, Guizhou, China; ^3^ Department of Laboratory Medicine, Kweichow Moutai Hospital, Zunyi, Guizhou, China

**Keywords:** *Vibrio cholerae*, DegS protease, aerobic metabolism, growth, intestinal colonization

## Abstract

Aerobic respiration is the key driver of *Vibrio cholerae* proliferation and infection. Our previous transcriptome results suggested that *degS* knockout downregulates a few genes involved in NADH and ATP synthesis in the aerobic respiratory pathway. In this study, non-targeted metabolomics results showed that the differential metabolites affected by *degS* knockout were associated with aerobic respiration. Further results suggested that the key products of aerobic respiration, NADH and ATP, were reduced upon *degS* deletion and were not dependent on the classical σ^E^ pathway. The two-component system response factor aerobic respiration control A (ArcA) is involved in regulating NADH and ATP levels. qRT-PCR demonstrated that DegS negatively regulates the transcription of the *arcA* gene, which negatively regulates the expression of isocitrate dehydrogenase (ICDH), a key rate-limiting enzyme of the tricarboxylic acid cycle. NADH and ATP levels were partially restored with the knockout of the *arcA* gene in the *ΔdegS* strain, while levels were partially restored with overexpression of ICDH in the *ΔdegS* strain. In a growth experiment, compared to the *ΔdegS* strain, the growth rates of *ΔdegSΔarcA* and *ΔdegS*-overexpressed *icdh* strains (*ΔdegS+icdh*) were partially restored during the logarithmic growth period. Colonization of the intestines of suckling mice showed a significant reduction in the colonizing ability of the *ΔdegS* strain, similar colonizing ability of the *ΔdegS::degS* strain and the wild-type strain, and a partial recovery of the colonizing ability of the *ΔdegS*+*icdh* strain. Overall, these findings suggest that the DegS protease regulates the expression of ICDH through ArcA, thereby affecting the NADH and ATP levels of *V. cholerae* and its growth and intestinal colonization ability.

## Introduction

*Vibrio cholerae* is a facultative anaerobic bacterium capable of both aerobic and anaerobic respiration (Reen et al., 2006). Bacteria produce chemical energy through aerobic-mediated energy metabolism, which is stored in the form of ATP to power the cellular processes required for growth. Both aerobic and anaerobic metabolisms are essential for the growth of *V. cholerae in vivo* (Bueno et al., 2020; Van Alst and DiRita, 2020). Aerobic respiration acts as a powerful driver of replication during infection with the *V. cholerae* gastrointestinal pathogen (Harris et al., 2012). In one study, *V. cholerae* incapable of aerobic respiration was strongly attenuated (10^5^ times) in young mice, whereas strains lacking anaerobic respiration showed no colonization defects (Van Alst et al., 2022). In a suckling mouse model, a related study reported that defects in the *pyruvate dehydrogenase* aerobic respiration gene of *V. cholerae* resulted in a significant decrease in colonization rates (Van Alst and DiRita, 2020). *In vitro*, *V. cholerae* undergoes aerobic respiration, which produces the metabolic intermediates succinate and pyruvate, resulting in increased motility (Kiiyukia et al., 1993). In addition, aerobic respiration promotes the transcription of the virulence factor *toxT* in *V. cholerae* during pathogenesis. Consequently, aerobic respiration plays a vital role in the pathogenicity of *V. cholerae* (Medrano et al., 1999; Fan et al., 2014). Concerning the control of cholera, it would be desirable to identify mechanisms that regulate aerobic respiration in *V. cholerae* and establish methods that can attenuate its effects.

The tricarboxylic acid (TCA) cycle is an important intermediate link in aerobic respiration and is regarded as the energy-generating engine of aerobic respiration for ATP synthesis. This function connects glycolysis and the electron transport chain and is a central part of cellular energy metabolism (MacLean et al., 2023). Aerobic respiration control A (ArcA) is a response factor in the two-component Arc system that acts as a global inhibitor of the aerobic respiratory pathway (particularly the TCA cycle), thereby promoting the bacterial fermentation pathway (Wang et al., 2018). Recently, a study demonstrated that overexpression of ArcA under aerobic conditions leads to downregulation of the respiratory pathway in *E. coli* (Basan et al., 2017). Hence, it is important to investigate the regulatory mechanisms of aerobic respiration in *V. cholerae* in terms of the global inhibitors of the TCA cycle.

Serine protease DegS is commonly recognized as an initiator of the σ^E^ (*rpoE*) stress response pathway (de Regt et al., 2015), which affects *V. cholerae* motility, chemotaxis and antioxidant capacity (Wang et al., 2023a; Zou et al., 2023). Our previous results by RNA sequencing (RNA-seq) showed that the knockout of *degS* resulted in the downregulation of genes associated with aerobic respiration, which are focused on the TCA cycle (Huang et al., 2019), but the mechanisms involved are not clear. In the current study, metabolomics analysis revealed that the differential metabolites of the *ΔdegS* mutant were mainly enriched in purine metabolism and glutathione metabolism associated with aerobic respiration, suggesting that DegS may regulate aerobic respiration in *V. cholerae.* This study investigated the influence of DegS on the key products of aerobic respiration, NADH, and ATP. Our results suggest that DegS affects isocitrate dehydrogenase (ICDH) expression through the regulation of ArcA, thereby affecting aerobic respiration in *V. cholerae*, which in turn affects NADH and ATP production.

## Materials and methods

### Bacterial strains and growth conditions

Non-O1/non-O139 *V. cholerae* HN375 from the China Center for Type Culture Collection (CCTCCAB209168) was used as the wild-type (WT) strain (Luo et al., 2011). Cloning was carried out using *Escherichia coli* DH5 and DH5-λpir, and conjugation were carried out using WM3064. Each strain was grown on Luria-Bertani (LB) medium at 37°C until the stationary phase was achieved unless otherwise indicated. The culture medium was modified by adding 0.1% arabinose and 100 g/mL ampicillin depending on the situation. Details of all plasmids and strains used are presented in **Supplementary Table 1**.

### DNA manipulations and genetic techniques

From the WT HN375 strain, deletion mutants were constructed with pWM91, a suicide plasmid (Wu et al., 2015). A list of the primers used can be found in **Supplementary Table 2**. To construct complementary mutants, the entire *arcA* encoding region by cloning into the pBAD24 plasmid vector, which was then transformed into *ΔdegSΔarcA* by electroporation to obtain *ΔdegSΔarcA:arcA*. A similar method was used to construct *ΔdegS*-overexpressed *icdh* strains (*ΔdegS+icdh*). As previously described, we used the pBAD24-*arcA* plasmid as a template and constructed a point mutation in D54E of *arcA* using site-directed mutagenesis (Fisher and Pei, 1997). The pBAD24-*arcA* ^D54E^ plasmid vector was then transformed into *ΔdegSΔarcA* by electroporation to obtain *ΔdegSΔarcA:arcA* ^D54E^. The complement and overexpression strains were grown in LB liquid medium with 0.1% arabinose for gene expression induction.

### Untargeted metabolomic analysis

The WT and *ΔdegS* strains were added into sterile LB liquid medium and shaken at 220 rpm and incubated at 37°C until the logarithmic growth phase (optical density at 600 nm [OD_600_] = 0.6). Both cultures were centrifuged at 10,000 g during 10 min at 4°C. After collection, the pellets were washed twice with 50 mM PBS, and used for untargeted metabolomic analysis. This analysis was performed by Biotech-Pack Scientific Co., Ltd. (Beijing, China). The Analysis Base File (ABF) converter software was used to convert the liquid chromatography-mass spectrometry (LC-MS) raw data into the ABF format (Zhang et al., 2021). The ABF format file was imported into MS-DIAL 4.10 software for preprocessing (D'Oria et al., 2022), including peak extraction, noise removal, inverse convolution, and alignment. The three-dimensional (3D) data matrix was exported in the CSV format (raw data matrix). Finally, the extracted peak information was searched against the MassBank, Respect, and Global Natural Product Social Molecular Networking (GNPS), for a full library comparison.

### Quantitative RT-PCR

All strains were grown to the stationary phase (OD_600_ = 1.2) in an LB liquid medium. Bacterial cultures were collected via centrifugation at 8000 rpm for 5 min. Total RNA was extracted with TRIzol reagent and reverse-transcribed into cDNA. qRT-PCR was performed using the TB Green Premix Ex TaqII (TaKaRa Bio, Shiga, Japan) (Huang et al., 2019). The 2^-ΔΔct^ method was used to calculate mRNA levels relative to each other (Livak and Schmittgen, 2001). For each qRT-PCR, two independent experiments were performed, each with three technical replicates.

### Assay of bacterial NADH levels

To examine the NADH levels of bacteria grown in LB liquid medium or M9 liquid medium (containing 0.4% glucose) to stationary phase (OD_600_ = 1.2), the concentration of bacteria was adjusted to approximately 1 × 10^8^ CFU/mL. The bacteria were ultrasonically lysed. NADH levels were measured using the Amplite™ Colorimetric NADH Assay Kit (AAT Bioquest, Pleasanton, CA, USA). Briefly, equal volumes of bacterial suspension and Amplite™ Colorimetric NADH Assay Kit working solution were mixed and dispensed in wells of a clear 96-well plate, followed by incubation at 26°C for 15 min to 2 h. Read the absorbance at 460 nm using an enzyme marker and construct a standard curve using the kit’s NADH standard (Xia et al., 2021). The experiment was repeated three times.

### Bacterial ATP levels assay

ATP levels were determined using an ATP Assay Kit (Beyotime, Shanghai, China) following the manufacturer’s instructions. Briefly, the bacteria were cultured to stationary phase in LB liquid medium or M9 liquid medium (containing 0.4% glucose) and the bacterial concentration was adjusted to 1 × 10^8^ CFU/mL. The bacteria were ultrasonically lysed. Equal volumes of the bacterial suspension and the working solution in the ATP reagent were mixed in a black opaque 96-well plate and incubated at 26°C for 5 min (Janet-Maitre et al., 2023). Luminescence was detected using a multifunctional enzyme labeler (Thermo Fisher Scientific Inc, Waltham, MA, USA). A standard curve was plotted using the standards provided in the kit. The experiment was repeated three times.

### Recombinant protein expression, purification, and preparation of polyclonal antisera

The His-tagged recombinant ArcA protein was constructed as previously described (Wang et al., 2023b). Briefly, primers were used to amplify the full-length ArcA-encoded open reading frames. The PCR product was ligated into the pET28a vector and transformed into *E. coli* BL21 (DE3). Transformed bacteria expressing ArcA were grown to OD_600_ = 0.6 at 37°C and induced with 0.5 mM isopropyl β-D-1-thiogalactopyranoside (IPTG) at 18°C for 16 h in LB liquid medium. Recombinant proteins labeled with His were purified by nickel-nitrilotriacetic acid affinity chromatography. Eight 6-week-old CD1 female mice, provided by the animal center of Zunyi Medical University (Zunyi, China), were housed in a specific pathogen-free environment and used to prepare anti-ArcA serum for western blot analysis. Mice were subcutaneously inoculated with 30 µg of recombinant ArcA on days 0, 14, and 28, along with the same volume of aluminum adjuvant. Anti-ArcA antiserum was prepared from blood collected 2 weeks after the last immunization.

### Western blot

All strains were grown to stationary phase (OD_600_ = 1.2) in an LB liquid medium. To prepare whole bacterial proteins, the culture was subjected to centrifugation, resulting in the separation of a supernatant layer. The precipitate was resuspended with 100 μL of distilled deionized water and 25 μL of 5× sodium dodecyl sulfate-polyacrylamide gel electrophoresis (SDS-PAGE) protein sampling buffer was added and boiled. The bacterial proteins were resolved by SDS-PAGE and transferred to a polyvinylidene fluoride membrane. After blocking, the membrane was incubated with a primary antibody (anti-ArcA serum at a dilution of 1:1000, prepared in our laboratory) overnight at 4°C. The membrane was incubated with a 1:5000 dilution of horseradish peroxidase-conjugated sheep anti-mouse IgG as a secondary antibody for 2 h after three washes with 1× Tris-buffered saline containing 0.1% Tween-20 (TBST). Finally, the membrane was washed three times with TBST, and color developed after the addition of chemiluminescent reagents (Epizyme Biomedical Technology Co. Ltd, Shanghai, China). The experiment was repeated three times.

### Bacterial growth curves

Growth curves were generated as described previously (Kovač et al., 2021) with certain modifications. Briefly, the bacteria were cultured in LB liquid medium at 37°C until the stationary phase (OD_600_ = 1.2). Aliquots of the culture were inoculated (1:500 v/v) into M9 liquid medium containing 0.4% glucose and incubated at 37°C with shaking at 200 rpm. Measurements were taken hourly for absorbance at 600 nm. The experiment was repeated three times.

### Suckling mouse colonization assay

Six-day-old CD1 suckling mice which were randomized into the experimental and control groups (n = 8 per group). All animal experiments were approved by the Ethics Committee of Zunyi Medical University (No. ZMU21-2301-069). All strains were grown at 37°C to stationary phase (OD_600_ = 1.2), and the bacteria were collected by centrifugation at 1,200 × *g* for 5 min. The bacterial concentration was adjusted to 1 × 10^7^ CFU/mL with PBS. Each suckling mouse in the experimental group was gavaged with 50 μL of the bacterial suspension. The same volume of 1× PBS was used in the negative control. In the 18th hour following gavage, mice were euthanized. The small intestinal tissue was subsequently dissected, weighed, and homogenized (Zou et al., 2023). After 100-fold dilution of this preparation, 100 μL aliquots were added to 0.5 mg/L gentamicin agar plates, and the colonies were counted after 18 h of incubation at 37°C. The final results are presented as the logarithm CFU/g.

### Statistical analyses

Data are expressed as mean ± standard deviation. Non-paired two-tailed t-tests were used to analyze differences between two groups, and a one-way analysis of variance was used to analyze differences between multiple groups. SPSS version 29.0 (IBM Corp., Armonk, NY, USA) was used for the analyses. *P*<0.05 indicated statistical significance.

## Results

### Non-targeted metabolomic analysis of the *degS* mutant

**Figure 1:**
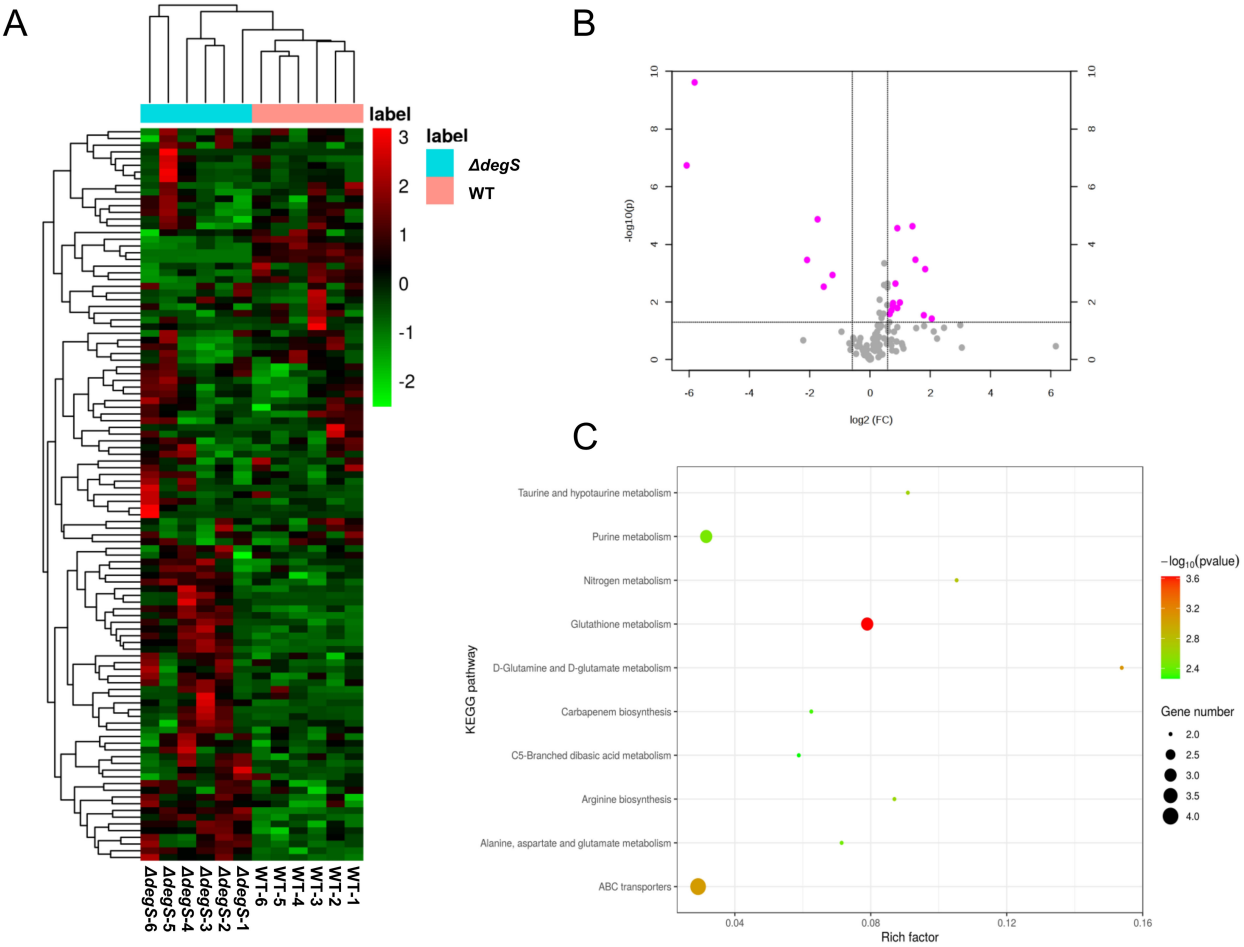
Non-targeted metabolomic analysis of the *degS* mutant. **(A)** Heatmap showing the abundance of 109 metabolites identified in the WT and *ΔdegS* strains. **(B)** Volcano plot depicting the identified metabolites in the WT and *ΔdegS* strains. The red dots represent significantly differentially expressed metabolites (fold change>2 or fold change<0.5, *P*<0.05). **(C)** Differentially metabolites were enriched in ten KEGG pathways.

Our previous RNA-seq data suggested that the knockout of *degS* results in the downregulation of genes related to the TCA cycle of the aerobic respiration pathway (Huang et al., 2019). The finding implies that DegS may affect aerobic respiration in *V. cholerae*. To further test this hypothesis, we conducted untargeted metabolomics on *degS* knockout mutants (*ΔdegS*). The analysis identified a total of 109 metabolites (**Figure 1A**; **Supplementary Table 3**). Significantly (*P*<0.05) differentially expressed metabolites were screened according to fold changes >2 or <0.5. A combination of multidimensional and unidimensional analyses identified 19 significantly differentially expressed metabolites (**Table 1**). One-dimensional statistical analyses were performed using multiplicity and t-tests. The resulting data were plotted as volcano plots (**Figure 1B**). Kyoto Encyclopedia of Genes and Genomes (KEGG) pathway enrichment analysis of enriched genes mainly revealed genes involved in purine and glutathione metabolism (**Figure 1C**). Glutathione metabolism is an important component of aerobic respiration and provides important redox buffers (Lushchak, 2012; Hatem et al., 2014). Purine metabolism provides purine nucleotides that are essential for ATP production from aerobic respiration (Gessner et al., 2023). These findings indicate that DegS has an impact on aerobic respiration in *V. cholerae*.

**Table 1:** Significantly differentially expressed metabolites of WT and *ΔdegS* strains.

Compounds	Log2 Fold Change (*ΔdegS/WT*)	-Log_10_ (*P* value)
9-methoxy-7-[4-[(2S,3R,4S,5S,6R)-3,4,5-trihydroxy-6-(hydroxymethyl)oxan-2-yl]oxyphenyl]-[1,3]dioxolo[4,5-g]chromen-8-one	-5.82	6.74
(4R)-3-methylidene-4-[(E)-3-methyl-4-(4-methyl-5-oxooxolan-2-yl)but-2-enyl]oxolan-2-one	-6.08	4.87
(E)-8-(4-hydroxy-6-methoxy-7-methyl-3-oxo-1H-2-benzofuran-5-yl)-2,6-dimethyloct-6-enoic acid	-1.74	4.63
(2S,6R,8aS)-6-(2-hydroxypropan-2-yl)-8a-methyl-4-methylidene-1,2,3,4a,5,6,7,8-octahydronaphthalen-2-ol-	1.41	4.56
Leupeptin	0.90	3.47
L-5-Oxoproline	1.50	3.46
Lenacil	-2.09	3.14
Falcarindiol	1.83	2.94
Glycine-Betaine	-1.25	2.64
DL-Coniine	0.84	2.53
Glycerophosphate(2)	-1.54	1.98
5-pentyl-2-furannonanoic acid	0.99	1.96
Tectorigenin	0.76	1.87
2-[2-(3,4-dimethoxyphenyl)ethyl]-4-methoxy-2,3-dihydropyran-6-one	0.76	1.79
Norharman	0.90	1.71
8-Prenylnaringenin	0.71	1.59
(3S)-5-[(1S,8aR)-2,5,5,8a-tetramethyl-4-oxo-4a,6,7,8-tetrahydro-1H-naphthalen-1-yl]-3-methylpentanoic acid	0.65	1.54
5-(1,2,4a,5-tetramethyl-7-oxo-3,4,8,8a-tetrahydro-2H-naphthalen-1-yl)-3-methylpentanoic acid	1.78	1.42
2’-Deoxyadenosine	2.05	4.87

### DegS positively affects NADH and ATP levels in *V. cholerae*

**Figure 2:**
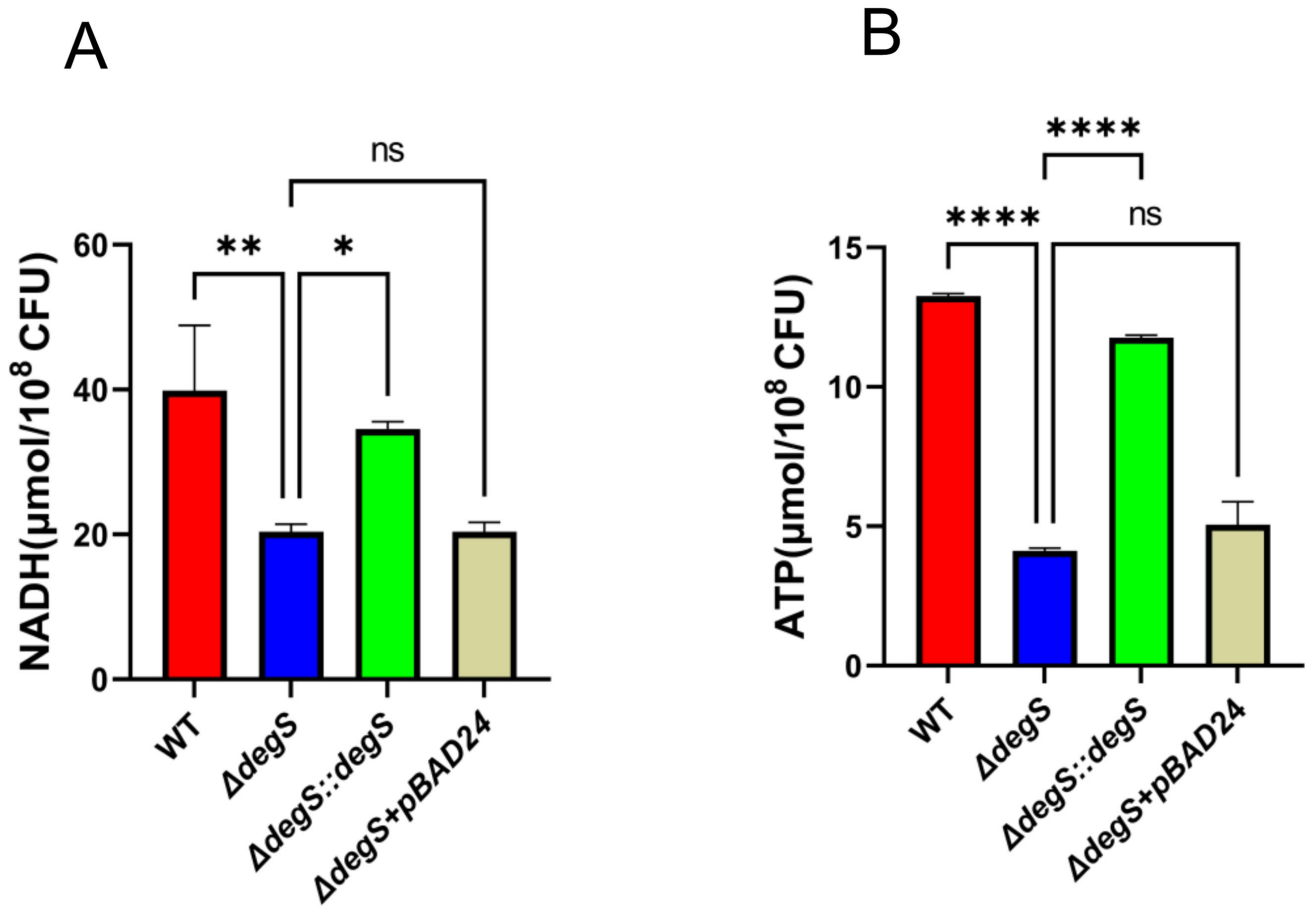
DegS positively affects NADH and ATP levels in *V. cholerae.* **(A)** Detection of NADH levels in wild type (WT), *ΔdegS*, *ΔdegS::degS*, and *ΔdegS+pBAD24*. **(B)** Assay of ATP levels in each strain. Data are expressed as the mean and standard deviation of biological replicates (n = 3). One-way analysis of variance (ANOVA) was employed for the analysis of the data. *, *P*<0.05; **, *P*<0.01; ****, *P*<0.0001; ns indicates no statistical significance.

Given the transcriptome and metabolome results, we hypothesized that DegS may affect the production of the aerobic respiration pathway products NADH and ATP. To test this hypothesis, we determined the levels of NADH in WT and *ΔdegS* strains. The NADH levels of the WT strain were approximately twice as high as those of the *ΔdegS* mutants, whereas the NADH levels of the complemented strain *ΔdegS::degS* were able to restore NADH levels close to those of the WT strain (**Figure 2A**). The pBAD24 null plasmid was unable to recover the NADH levels of *ΔdegS*. Subsequently, we investigated ATP levels in the above strains, which is the final energy product of the aerobic respiration pathway. The ATP level of the WT strain was approximately thrice that of *ΔdegS*, whereas the ATP levels of *ΔdegS::degS* were partially restored, with no restoration using the pBAD24 empty plasmid (**Figure 2B**). The above data suggest that DegS influences positively NADH and ATP levels in *V. cholerae.*

### DegS positively affects NADH and ATP levels in *V. cholerae* independent of σ^E^

**Figure 3:**
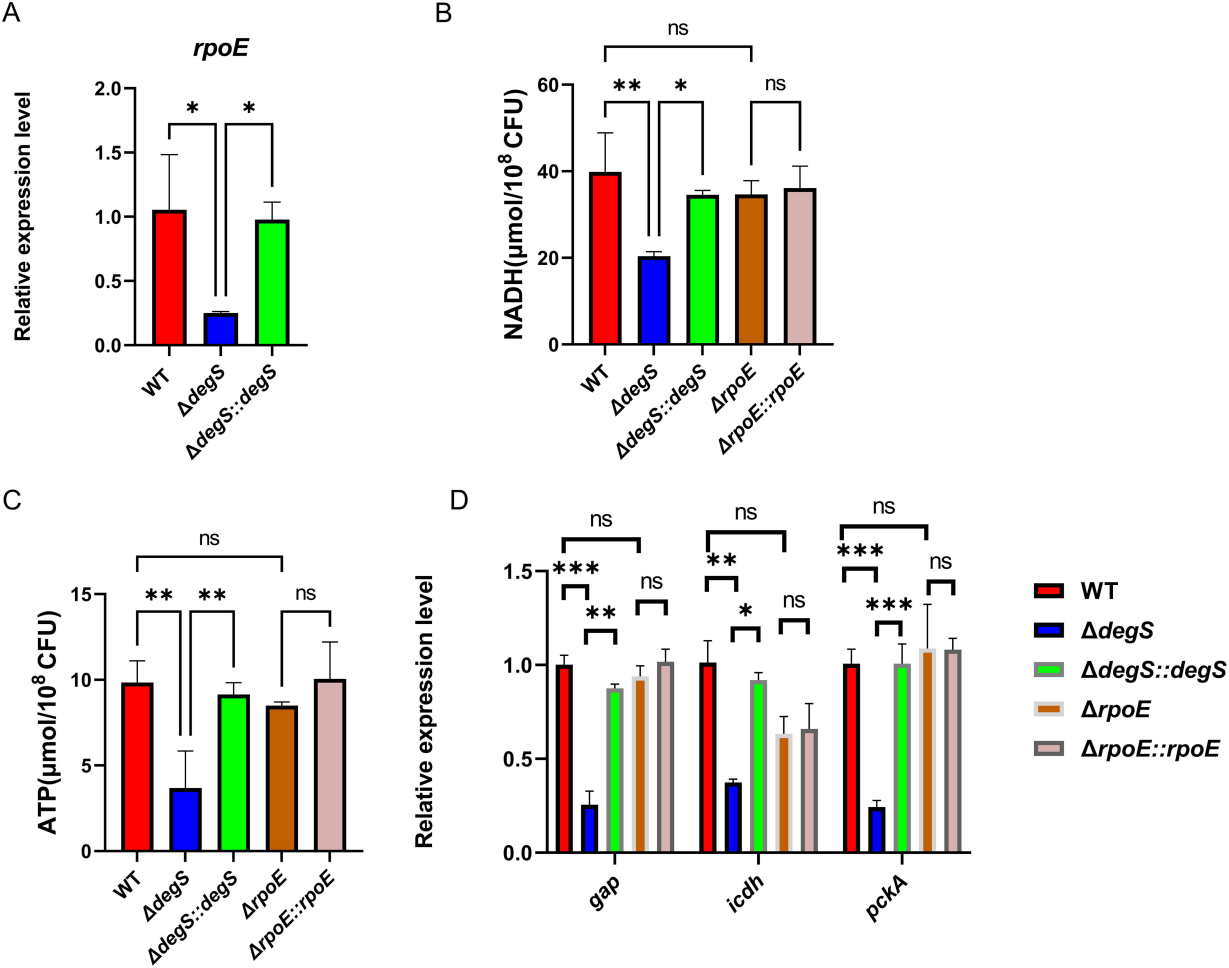
DegS positively affects NADH and ATP levels in *V. cholerae* independent of σ^E^. **(A)** The mRNA levels of *rpoE* in the WT, *ΔdegS*, and *ΔdegS::degS.* **(B, C)** Detection of NADH **(B)** and ATP **(C)** levels in WT, *ΔdegS*, *ΔdegS::degS*, *ΔrpoE*, and *ΔrpoE::rpoE*. **(D)** The mRNA levels of aerobic respiration-related genes in each strain. Analyses were performed using the one-way ANOVA statistical method. The data are presented as the mean and standard deviation of each of three biological replicates. (n = 3). *, *P*<0.05; **, *P*<0.01; ***, *P*<0.001; ns indicates no statistical significance.

DegS regulates stress response and motility through σ^E^ and DegS deficiency significantly reduces σ^E^ activity (Ades et al., 1999). To investigate whether DegS affects *V. cholerae* NADH and ATP levels via σ^E^, we first examined the transcript levels of *rpoE*. The qRT-PCR results showed that the *rpoE* gene transcript level in the WT strain was approximately four times higher than that of *ΔdegS*, while the transcript level of the *rpoE* gene in *ΔdegS::degS* was almost the same as that of the WT strain (**Figure 3A**). Next, we performed experiments for the detection of NADH and ATP levels using the *rpoE* deletion mutant (*ΔrpoE*) and corresponding complemented strain (*ΔrpoE::rpoE*). The NADH level of the *ΔrpoE* mutant was not statistically different from the WT and *ΔrpoE::rpoE* strains (**Figure 3B**). The ATP levels of the *rpoE* mutation did not differ from that of the WT and *ΔrpoE::rpoE* strains (**Figure 3C**). We used qRT-PCR to screen for changes in the expression of some aerobic respiratory genes in different strains, and the expression levels of genes encoding type I glyceraldehyde-3-phosphate dehydrogenase (GAP), isocitrate dehydrogenase (ICDH), and phosphoenolpyruvate carboxykinase (PckA) were significantly reduced in the *ΔdegS* mutant as compared with the WT strain (**Figure 3D**). However, the expression of the above genes did not change after *rpoE* knockout. These results indicate that DegS positively affects NADH and ATP levels in *V. cholerae* independent of σ^E^.

### Effect of DegS on NADH and ATP levels in *V. cholerae* involves ArcA

**Figure 4:**
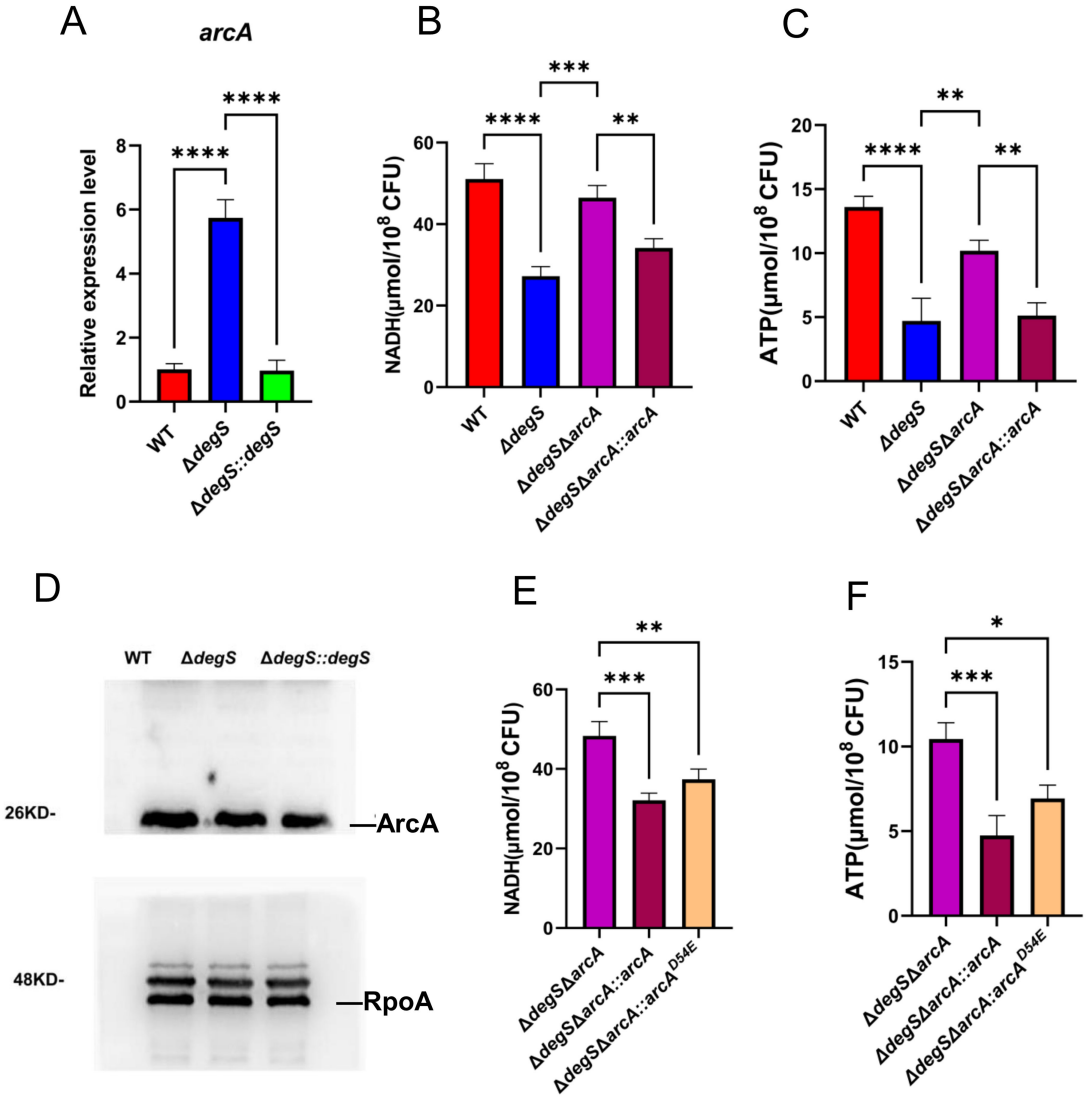
Effect of DegS on NADH and ATP levels in *V. cholerae* involves ArcA. **(A)** The mRNA levels of *arcA* in different strains. **(B, C)** Detection of NADH **(B)** and ATP **(C)** levels in WT, *ΔdegS*, *ΔdegSΔarcA*, and *ΔdegSΔarcA::arcA*. **(D)** Western blot analysis of the whole bacterial proteins of WT, *ΔdegS*, and *ΔdegS::degS* strains using anti-ArcA serum. **(E, F)** Detection of NADH **(E)** and ATP **(F)** levels in *ΔdegSΔarcA*, *ΔdegSΔarcA::arcA*, and *ΔdegSΔarcA::arcA^D54E^.* These values are expressed as the mean and standard error of three biological replicates (n = 3) and are subjected to one-way analysis of variance (ANOVA) for analysis. *, *P*<0.05; **, *P*<0.01; ***, *P*<0.001; ****, *P*<0.0001; ns means no statistical significance.

In *S. typhimurium*, ArcA may negatively regulate ATP and NADH levels by inhibiting gene transcription levels of the pyruvate dehydrogenase complex (PDH) in the TCA cycle (Morales et al., 2013). Using qRT-PCR, we observed that the transcript level of *arcA* in *ΔdegS* was approximately six times higher than that of the WT strain (**Figure 4A**), suggesting that DegS is a negative regulator of ArcA. Therefore, we hypothesized that DegS influences the NADH and ATP levels in *V. cholerae* through ArcA. To assess the hypothesis, we constructed *ΔdegSΔarcA* and *ΔdegSΔarcA::arcA* and measured the levels of NADH and ATP. Both levels in *ΔdegSΔarcA* could be partially restored compared to *ΔdegS* (**Figures 4B, C**). Next, we detected the expression level of ArcA protein in each strain. ArcA protein expression was almost the same in WT strains, *ΔdegS*, and *ΔdegS::degS* (**Figure 4D**).ArcA is a response factor in a two-component system that can activate downstream genes in a phosphorylated form. Meanwhile, in *E. coli*, ArcA is an important inhibitor, and its phosphorylated form directly inhibits the expression of some genes in the TCA cycle, such as citrate synthase (GltA) and malate dehydrogenase (MDH) (Park et al., 2013). Therefore, we speculated whether its phosphorylation modifications are involved in this regulatory process. Next, we constructed a point mutation model (*ΔdegSΔarcA::arcA^D54E^*) to mimic dephosphorylation (Jeon et al., 2001) to explore whether ArcA phosphorylation is associated with DegS affecting NADH and ATP levels in *V. cholerae*. Both NADH and ATP levels were decreased in the *ΔdegSΔarcA::arcA^D54E^* strain compared to the *ΔdegSΔarcA* strain. The *ΔdegSΔarcA::arcA^D54E^* strain had a smaller decrease in NADH and ATP levels than the *ΔdegSΔarcA::arcA* strain (**Figures 4E, F**). These results suggest that DegS affects NADH and ATP levels, which are partially dependent on ArcA phosphorylation.

### Effect of DegS on NADH and ATP levels in *V. cholerae* involved in expressing ICDH

**Figure 5:**
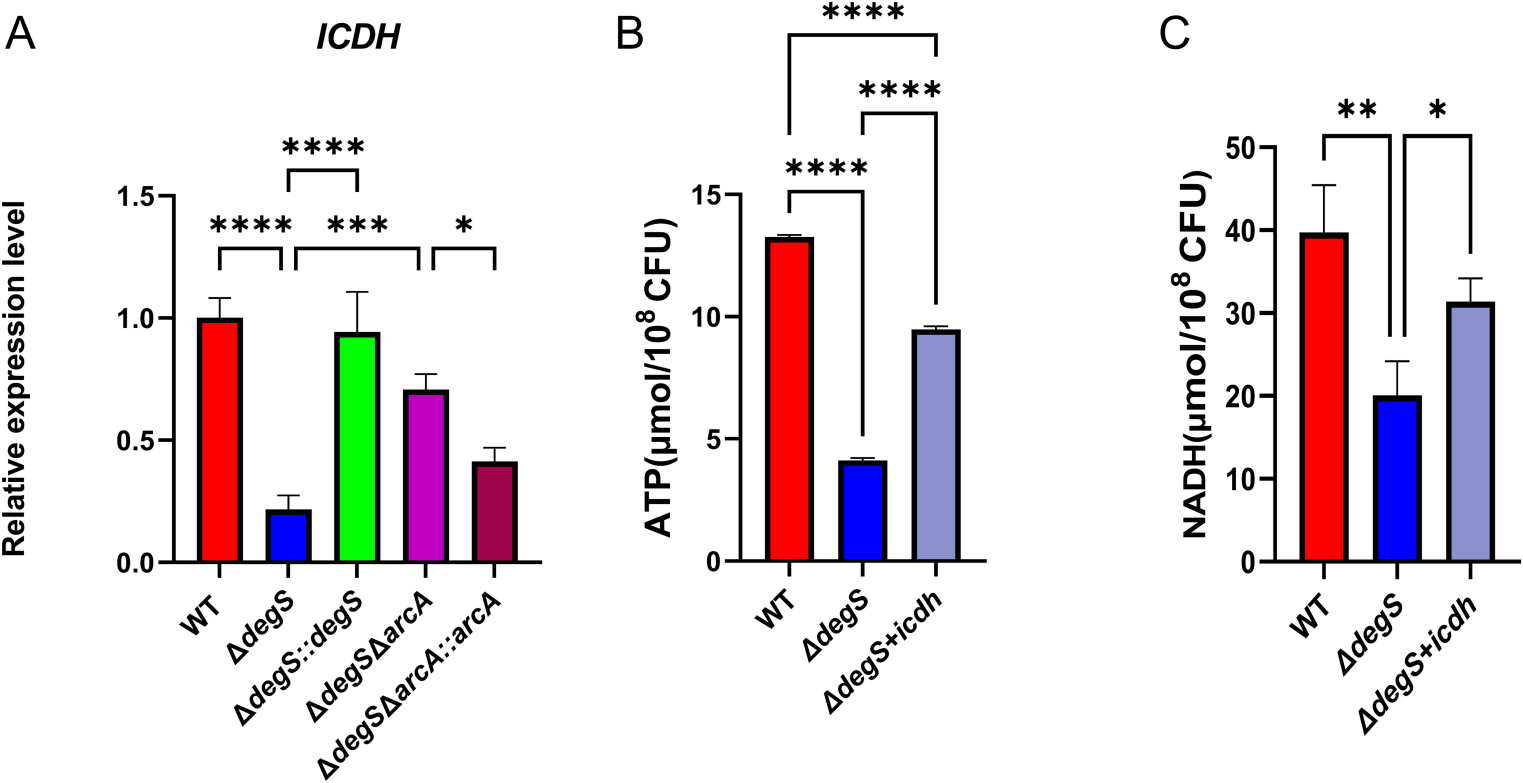
Effect of DegS on NADH and ATP levels in *V. cholerae* involved in the expression of ICDH. **(A)** The mRNA levels of *icdh* in WT, *ΔdegS*, *ΔdegS::degS*, *ΔdegSΔarcA*, and *ΔdegSΔarcA::arcA* strains. **(B, C)** Detection of ATP **(B)** and NADH **(C)** levels in WT, *ΔdegS*, and *ΔdegS*+*icdh*. Data are expressed as mean and standard deviation of three biological replicates (n = 3) and were analyzed using one-way ANOVA. *, *P*<0.05; **, *P*<0.01; ***, *P*<0.001;****, *P*<0.0001.

ICDH is a key rate-limiting enzyme of the TCA cycle; the knockdown of ICDH leads to a decrease in bacterial NADH and ATP levels (Kabir and Shimizu, 2004a). Our qRT-PCR results revealed that the transcription level of *icdh* in *ΔdegS* strains was approximately five times lower than that of WT strains and that the transcriptional level of *icdh* was recovered in part in the *ΔdegSΔarcA* strain (**Figure 5A**). These findings suggest that DegS may control the transcription of *icdh* through the ArcA pathway. To determine whether ICDH is involved in regulating *V. cholerae* NADH and ATP levels in DegS, we overexpressed ICDH based on the *ΔdegS* strain and measured NADH and ATP levels. Both levels were partially restored in the *ΔdegS+icdh* strain, but not to the level of the WT strain (**Figures 5B, C**). Collectively, these results suggest that DegS is required for high levels of ATP and NADH because it indirectly increases ICDH expression.

### DegS affects the growth of *V. cholerae* through the ArcA-ICDH pathway

**Figure 6:**
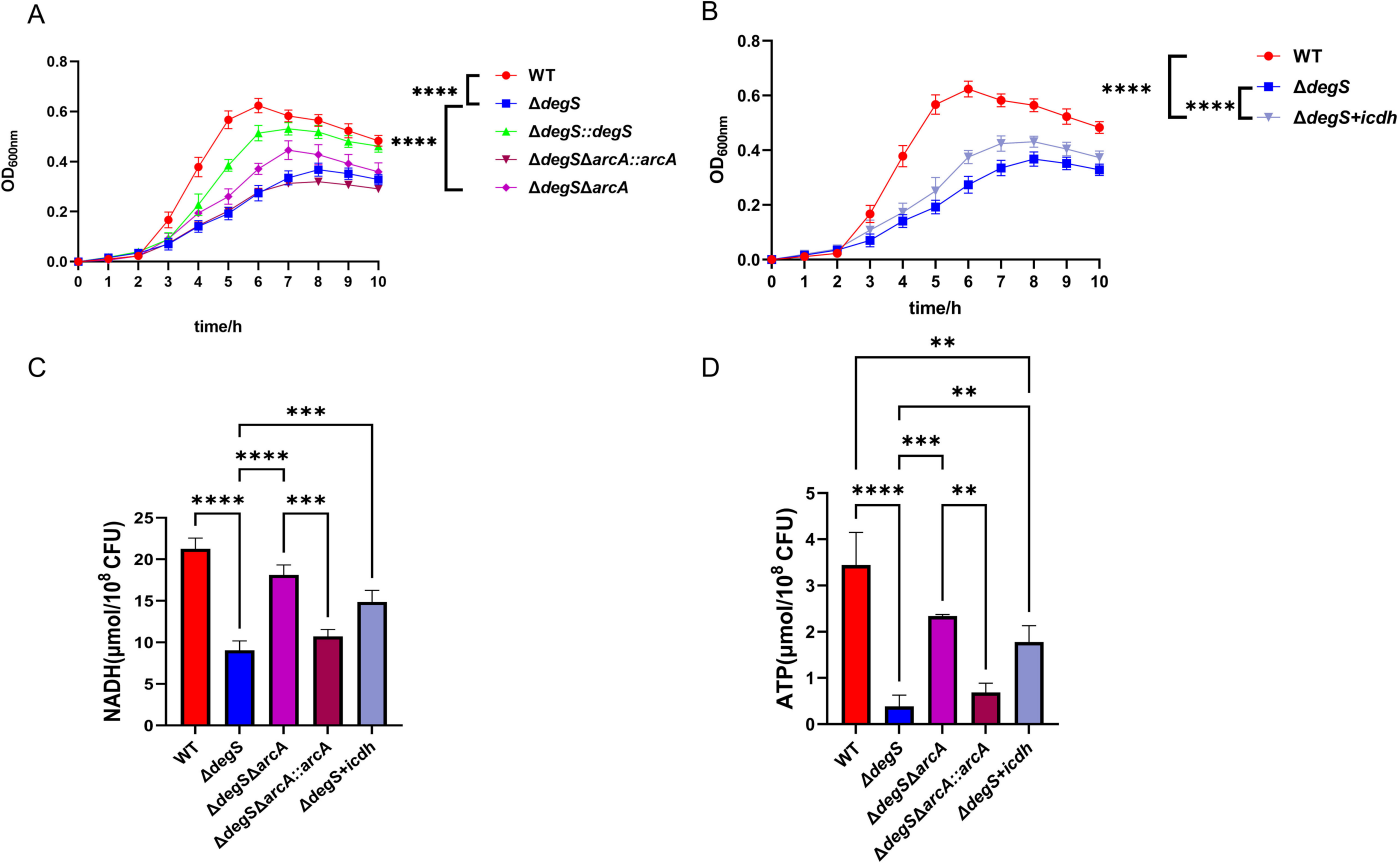
DegS affects the growth of *V. cholerae* through the ArcA-ICDH pathway. **(A, B)** Growth curves of WT, *ΔdegS*, *ΔdegS::degS*, *ΔdegSΔarcA*, *ΔdegSΔarcA::arcA*, and *ΔdegS*+*icdh* strains in M9 medium with 0.4% glucose added at 37°C. **(C, D)** Detection of NADH **(C)** and ATP **(D)** levels of WT, *ΔdegS*, *ΔdegSΔarcA*, *ΔdegSΔarcA::arcA*, and *ΔdegS*+*icdh* strains in M9 medium. The values are shown as mean and standard deviation of three biological replicates (n = 3) and were analyzed using one-way ANOVA. **, *P*<0.01; ***, *P*<0.001; ****, *P*<0.0001.

Many enzymes and metabolites associated with bacterial energy metabolism have direct regulatory roles in bacterial growth (Kabir and Shimizu, 2004a; Weart et al., 2007; Hill et al., 2013; Sperber and Herman, 2017). Our experiments showed that DegS affects ATP and NADH levels in *V. cholerae* through the ArcA-ICDH signaling pathway. To confirm whether DegS affects growth in *V. cholerae* through this pathway, we conducted growth curve experiments in the M9 medium. The growth rate of the *ΔdegS* strain was lower than that of the WT strain during the logarithmic growth phase (**Figure 6A**). Compared to the *ΔdegS* strain, the *ΔdegSΔarcA* strain grew faster during the logarithmic growth period. The *ΔdegS*+*icdh* strain had a faster growth rate than the *ΔdegS* strain during the logarithmic growth phase (**Figure 6B**). Concurrently, the trends of NADH and ATP levels of the *ΔdegSΔarcA* strain and the *ΔdegS*+*icdh* strain in M9 medium corresponded to the trends of their growth rates in the logarithmic growth phase (**Figures 6C, D**). These evidences demonstrate that DegS affects the growth of *V. cholerae* through the ArcA-ICDH pathway.

### DegS affects *V. cholerae* intestinal colonization

**Figure 7:**
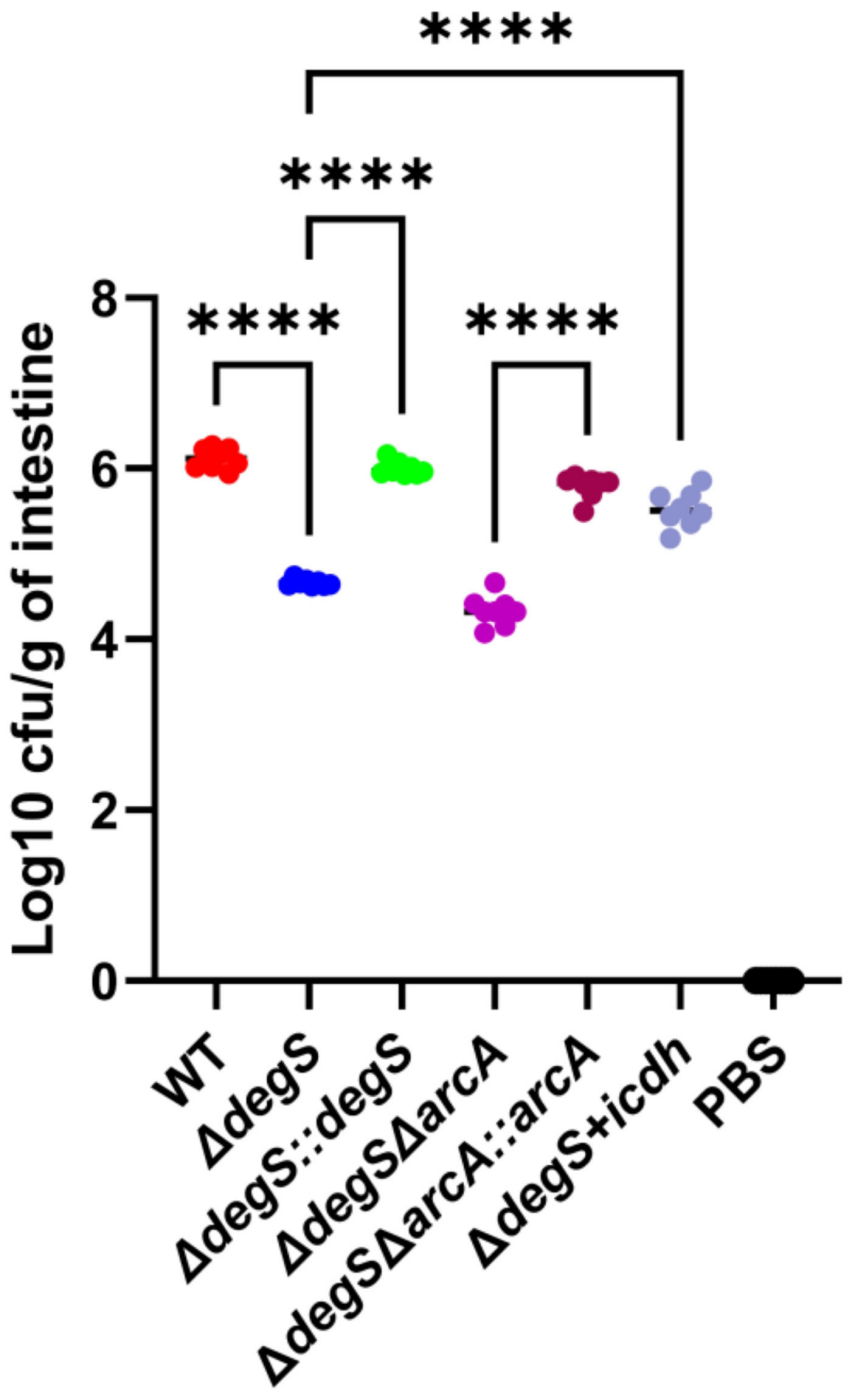
DegS affects *V. cholerae* intestinal colonization. Approximately 10^7^ cells of different strains were gavaged into suckling mice. The results obtained after 18 h are expressed as the logarithm of colony-forming units/g intestine (CFU/g; mean± SD, n = 8). Values were analyzed by one-way ANOVA, ****, *P*<0.0001.

Inhibition of NADH and ATP production in bacteria affects their colonization (Jones et al., 2007; Schurig-Briccio et al., 2020). To examine whether the regulation of *V. cholerae* NADH and ATP levels mediated by DegS is critical for bacterial colonization, we used a suckling mouse model of intestinal colonization. The *in vivo* results showed that compared with the WT strain, the colonization capacity of the *ΔdegS* strain was significantly reduced, while the colonization capacity of the *ΔdegS::degS* strain was similar to that of the WT strain, and the colonization ability of the *ΔdegS+icdh* strain was partially restored (**Figure 7**). However, the colonization ability of *ΔdegSΔarcA* was comparable to that of *ΔdegS*, and the colonization ability of *ΔdegSΔarcA::arcA* was stronger than that of *ΔdegSΔarcA* strain.

## Discussion

**Figure 8:**
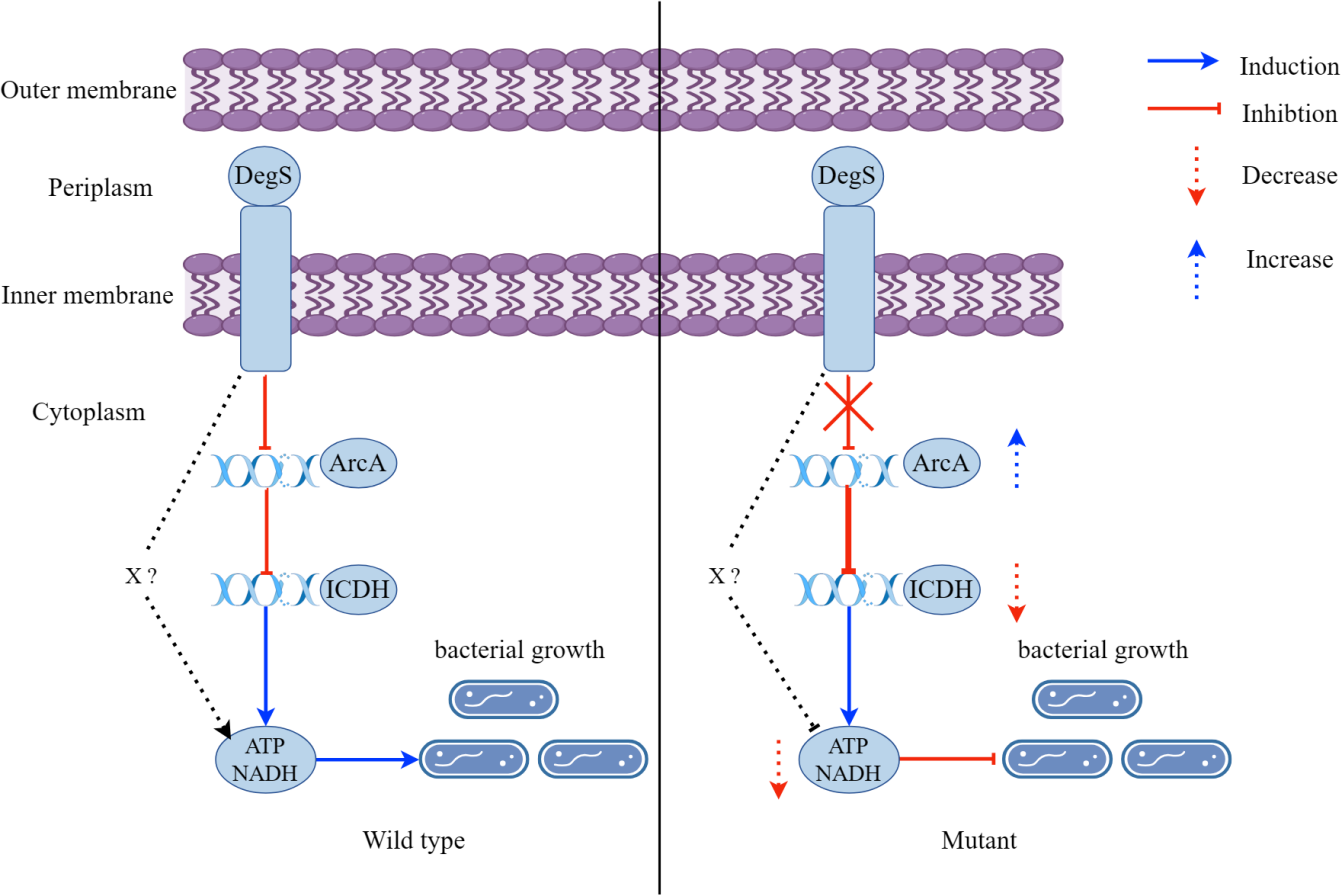
A model showing that DegS positively regulates ATP and NADH levels to promote the growth of *V. cholerae*, which is in part dependent on the ArcA-ICDH pathway. A red arrow represents inhibition and a downward-pointing dashed red arrow represents a reduction. A blue arrow represents induction and an upward-pointing blue dashed arrow represents an increase.

Aerobic respiration is a major driver of *V. cholerae* proliferation during infection. *V. cholerae* require energy from aerobic respiration for subsequent proliferation and infection (Van Alst and DiRita, 2020). Here, we observed that DegS protease plays a vital role in NADH and ATP levels, growth, and colonization of *V. cholerae*. We propose a model whereby DegS positively regulates ATP and NADH levels to promote the growth of *V. cholerae*, which is in part dependent on the ArcA-ICDH pathway. In addition, there may be other factors (X) involved in the effects of DegS on *V. cholerae* NADH and ATP levels, and growth (**Figure 8**).

The DegS serine protease is located within the bacterial periplasm. The protein is thought to be involved in initiating the σ^E^ stress response pathway, where active DegS catalyzes the cleavage of RseA, releasing active σ^E^, which activates σ^E^-regulated gene expression (Sohn et al., 2007; Chaba et al., 2011). Although σ^E^ is involved in a variety of biological processes, such as stress response, biofilm formation, and motility (Liang et al., 2021), its relevance to aerobic respiration has remained unclear. Our study reveals that the levels of NADH and ATP, which are key products of aerobic respiration, decreased upon deletion of *degS* (**Figure 2A, B**). However, deletion of *rpoE* had little effect on NADH and ATP levels (**Figures 3B, C**). In addition, qRT-PCR results revealed no statistically significant changes in any of the relevant aerobic respiration genes in the *ΔrpoE* strain (**Figure 3D**). Given these results, we speculate that the effect of DegS on *V. cholerae* NADH and ATP levels is independent of σ^E^. This suggests that DegS may have a different pathway than the previous dependence on σ^E^.

Further investigating the mechanism by which DegS affects NADH and ATP levels in *V. cholerae*, we observed using RNA-seq that deletion of *degS* mainly inhibits the TCA cycle, carbon metabolism, and pyruvate metabolism (Huang et al., 2019). Meanwhile, qRT-PCR results showed that aerobic respiratory-related genes were altered in the *ΔdegS* mutant (**Figure 3D**). Among them, the expression of the *gap* gene, which is a key enzyme involved in glycolysis, was significantly reduced. Expression of the *pckA* gene, which is involved in gluconeogenesis, was also reduced. Notably, the expression of the *icdh* gene, a key gene in the TCA cycle, was significantly reduced. Since the TCA cycle is a major biochemical hub in most heterotrophic organisms, it is essential for aerobic respiration (Brandenburg et al., 2021; Jiang et al., 2023). Therefore, we chose *icdh* to further investigate the mechanism by which DegS affects NADH and ATP levels in *V. cholerae*.

ArcA acts as a response factor in a two-component system to directly or indirectly inhibit the TCA cycle, thereby reshuffling bacterial metabolic pathways and optimizing energy conversion (Gunsalus and Park, 1994; Liu and De Wulf, 2004; Brown et al., 2023). ArcA as a global transcription factor responds to NADH and ATP (Holm et al., 2010). In addition, the *ΔarcA* strain of *Salmonella enterica* displays higher levels of NADH (Morales et al., 2013). In this study, we observed that an increase in transcript levels of *arcA* after knockout of *degS* (**Figure 4A**) and knockout of the *arcA* gene partially restored the low levels of NADH and ATP levels in the *ΔdegS* strain (**Figures 4B, C**). At the protein level, western blot experiments revealed no difference in ArcA protein expression in the *ΔdegS* strain (**Figure 4D**). Therefore, we speculate that the post-translational modification of ArcA may be involved in the regulation of NADH and ATP by DegS. ArcA can be activated as a transcription factor via phosphorylation to regulate the expression of downstream genes (Yan et al., 2021). Therefore, suspecting that ArcA may play a role in phosphorylation, we constructed a model of dephosphorylation by point mutation (*ΔdegSΔarcA::arcA^D54E^*). Both NADH and ATP levels were lower significantly in the point mutant strain compared to the *ΔdegSΔarcA* strain, but not as much as in the *ΔdegSΔarcA::arcA* strain (**Figures 4E, F**). Thus, we speculated that DegS affects NADH and ATP levels is partially dependent on ArcA phosphorylation. This phenomenon is similar to the EnvZ/OmpR two-component system in *Klebsiella pneumoniae*, where the *ΔompR* mutant completely loses mucoviscosity compared to the wild-type strain, while the unphosphorylated *ompR* ^D55A^ mutant reduces mucoviscosity only to a lesser extent, suggesting that phosphorylation only partially affects its phenotype (Wang et al., 2023b). Brown et al. show that the conserved metabolic regulator ArcA responds to host-mediated cell envelope damage (Brown et al., 2023). Meanwhile, DegS is a serine protease that mediates the cell envelope stress response, so we hypothesized that they might be linked through the cell envelope stress response pathway.

ICDH is one of the vital rate-limiting enzymes in the TCA cycle (Krebs and Johnson, 1980; Cronan and Laporte, 2005) and its transcription is dependent on ArcA (Chao et al., 1997). In addition, knockout of *icdh* in the TCA cycle results in changes in the central metabolism of *E. coli*, such as a decrease in intracellular NADH and ATP levels and a decrease in the rate of glucose consumption (Kabir and Shimizu, 2004a). In the current study, qRT-PCR showed that DegS positively regulated *icdh*, and ArcA negatively regulated the *icdh* gene (**Figure 5A**). Overexpression of ICDH partially restored NADH and ATP levels in the *ΔdegS* strain (**Figures 5B, C**). Based on these results, we suggest that DegS affects NADH and ATP levels in *V. cholerae* via ArcA, in relation to ICDH.

Bacteria require energy to grow, and a decrease in the energy supply can inhibit their growth (Orellana et al., 2022; Ren et al., 2022). ATP and NADH are important components of the energy supply and are essential for bacterial growth. Previous studies demonstrated that ArcA affects bacterial NADH levels and growth (Xie et al., 2021; Yan et al., 2021). In addition, deletion of *E. coli icdh* leads to alterations in NADH and ATP levels, thereby affecting specific growth (Kabir and Shimizu, 2004a). Here, we observed that the trends in NADH and ATP levels of the *ΔdegSΔarcA* and *ΔdegS+icdh* strains in the M9 medium (**Figure 6C, D**) were consistent with the same growth rate trends in the logarithmic growth phase (**Figures 6A, B**). Therefore, we propose that DegS regulates *V. cholerae* NADH and ATP levels through the ArcA-ICDH pathway, thereby affecting *V. cholerae* growth. Given that the *ΔdegSΔarcA* strain and the *ΔdegS+icdh* strain only partially restored the growth rate of *ΔdegS* in the logarithmic growth phase, NADH and ATP levels were not fully restored in the *ΔdegS+icdh* strain, we speculate that DegS regulation of *V. cholerae* growth and the levels of ATP and NADH may involve other factors.

Energy is an important driver of bacterial colonization (Chandrashekhar et al., 2018). We observed a highly significantly reduced colonization ability in the *ΔdegS* mutant, consistent with our previous study (Zou et al., 2023). However, the colonization ability of *ΔdegSΔarcA* was not restored to a certain extent. This result may be due to the fact that *arcA* is required for *V. cholerae* biofilm formation (Xi et al., 2020), which is important for intestinal colonization (Silva and Benitez, 2016). Compared to the *ΔdegSΔarcA* strain, the *ΔdegS+icdh* strain are directly overexpressing the *icdh* gene and do not involve the knockout of the *arcA* gene, so their colonization ability can be partially restored.

## Conclusion

In summary, we demonstrate that deletion of *degS* leads to a decrease in NADH and ATP levels in *V. cholerae* and is independent of σ^E^, thus inhibiting *V. cholerae* growth. Furthermore, our findings indicated that DegS may be a prospective mechanism for regulating ICDH expression through ArcA. These findings enhance the knowledge of the biological functions of DegS and offer new perspectives on the regulation of NADH and ATP levels in *V. cholerae*. The role of DegS in ICDH regulation through ArcA independent of σ^E^ needs to be further investigated.

## Data Availability

The datasets presented in this study can be found in online repositories. The names of the repository/repositories and accession number(s) can be found in the article/**Supplementary Material**.
