# Supplementary material for: DegS regulates the aerobic metabolism of Vibrio cholerae via the ArcA-isocitrate dehydrogenase pathway for growth and intestinal colonization
Source: Front Cell Infect Microbiol. 2024 Nov 1;14:1482919. doi: 10.3389/fcimb.2024.1482919 (PMC11564185; doi:10.3389/fcimb.2024.1482919)
Supplement: Supplementary file 1 [file Table1.docx]

Supplementary Material

**Supplementary Table 1 The information of bacterial strains and plasmids used in this study**

| **Strains or plasmids** | **Genotype or feature(s)** | **Reference or source** |
| --- | --- | --- |
| **Strains** |  |  |
| Non-O1/non-O139 *Vibrio cholerae* Strains |  |  |
| HN375 | Wild-type non-O1/non-O139 *V. cholerae* | Lab collections |
| *ΔdegS* | in-frame deletion of *degS* | Lab collections |
| *ΔdegS::degS* | *ΔdegS* complemented with pBAD24-*degS*, Amp^r^ | Lab collections |
| *ΔdegS*+pBAD24 | *ΔdegS* complemented with pBAD24, Amp^r^ | Lab collections |
| *ΔrpoE* | in-frame deletion of *rpoE* | Lab collections |
| *ΔrpoE::rpoE* | *ΔrpoE* complemented with pBAD24-*rpoE*, Amp^r^ | Lab collections |
| *ΔdegSΔarcA* | in-frame deletion of *degS* and *arcA* | This study |
| *ΔdegSΔarcA::arcA* | *ΔdegSΔarcA* complemented with pBAD24-*arcA*, Amp^r^ | This study |
| *ΔdegSΔarcA::arcA^D54E^* | *ΔdegSΔarcA* complemented with pBAD24-*arcA^D54E^*, Amp^r^ | This study |
| *ΔdegS*+*icdh* | *ΔdegS* complemented with pBAD24-*icdh*, Amp^r^ | This study |
| *E. coli* Strains |  |  |
| DH5α | F−, ø80dlacZ*Δ*M15, *Δ*(lacZYA-argF) U169 deoR, recA1, endA1, hsdR17 (rk-,mk+), phoA, supE44, ʎ-, thi-1, gyrA96, relA1 | Lab collections |
| DH5α( λpir) | supE44 *Δ*lacU169 (ΦlacZ*Δ*M15) recA1 endA1 hsdR17 thi-1 gyrA96 relA1 λpir | Lab collections |
| WM3064 | thrB1004 pro thi rpsL hsdS lacZ*Δ*M15 RP4-1360 *Δ*(araBAD)567 *Δ*dapA1341::[erm pir] | Lab collections |
| BL21（DE3） | F-, lon-11, *Δ*(ompT-nfrA)885, *Δ*(galM-ybhJ)884, λDE3[lacI lacUV5-T7 gene 1 ind1 sam7 nin5], *Δ*46, [mal+]K-12(λS), hsdS10 | Lab collections |
| pET28a-*arcA*-BL21（DE3） | BL21 (DE3) complemented with pET28a-*arcA*, Kan^r^ | This study |
| **Plasmids** |  |  |
| pWM91 | Suicide plasmid; oriR oriT lacZ tetAR sacB, Amp^r^ | Lab collections |
| pWM91-*ΔarcA* | pWM91 carrying upstream and downstream fragments flanking *arcA*, Amp^r^ | This study |
| pBAD24 | Expression vector with araBAD promoter and rrnB T1 terminator, Amp^r^ | Lab collections |
| pBAD24-*arcA* | pBAD24 expressing ArcA, Amp^r^ | This study |
| pBAD24-*arcA^D54E^* | pBAD24 expressing ArcA^D54E^, Amp^r^ | This study |
| pBAD24-*icdh* | pBAD24 expressing ICDH, Amp^r^ | This study |
| pET28a | Expression vector with N-6×His, N-Thrombin, N-T7, C-6×His tag, Kan^r^ | Lab collections |
| pET28a-*arcA* | pET28a expressing ArcA tagged with 6×His, Kan^r^ | This study |

**Supplementary Table 2 The list of the primers used in the study**

| **Name** | | **Primer sequence (forward/reverse, 5’ to 3’)** | **Use and description** |
| --- | --- | --- | --- |
| Primers for mutant construction | | | |
| *arcA* up-F | | CGCGGATCCCTCATCAACACAAGAACAGAG | For construction of deletion mutant of *ΔdegSΔarcA* |
| *arcA* up-R | | ATCGTTACATCAACAGTACGTGCGGGGTTTGCATTAGCGTT |  |
| *arcA* down-F | | AACGCTAATGCAAACCCCGCACGTACTGTTGATGTAACGAT |  |
| *arcA* down-R | | CCGCTCGAGGCTGCAATCCTATGTTGAGA |  |
| Primers for constructs for complementation | | | |
| *arcA* -F | | CCGGAATTCATGCAAACCCCGCAGATCCT | For cloning complete length of *arcA* into pBAD24 |
| *arcA* -R | | CCCAAGCTTTTAATCTTCTAAATCACCAC |  |
| *icdh* -F | | CCGGAATTCATGCCTACCAATAAACCTACAAT | For cloning complete length of *icdh* into pBAD24 |
| *icdh* -R | | CCCAAGCTTTTAACGGTCAATGATCGCGTT |  |
| Primers for construction for point mutation | | | |
| ArcA-D54E-F | CTTGGTGATTATGGAAATCAACCTGCCAGG | | For construction of ArcA mutant D54E in pBAD24 |
| ArcA-D54E-R | CCTGGCAGGTTGATTTCCATAATCACCAAG | |  |
| Primers for Real time PCR | | | |
| q*-gap-*F | | CCGCATTAGACGCCGCACTAC | For qRT-PCR to targeting *gap* |
| q*-gap-*R | | TCCACCACGCACAACAATCGC |  |
| q-*arcA-*F | | ATGCGGGCACCACACAAGAAG | For qRT-PCR to targeting *arcA* |
| q*-arcA-*R | | CAGCAGAGCACGGAACTCAGAAC |  |
| q*-icdh-*F | | TCTGGCGATGGTGGACTCTGAC | For qRT-PCR to targeting *icdh* |
| q*-icdh-*R | | AGTACGGATCATTGCTGGCATAGAC |  |
| q-*pckA*-F | | GAGCGTGGCATTACTGAACCTACC | For qRT-PCR to targeting *pckA* |
| q-*pckA*-R | | CGCTGCCGTTCCAACCTGTG |  |
| q-16sRNA-F | | CGGTAATACGGAGGGTGCAA | For qRT-PCR to targeting 16sRNA |
| q-16sRNA-R | | CACCTGCATGCGCTTTACG |  |
